# Better oral hygiene is associated with a reduced risk of osteoporotic fracture: a nationwide cohort study

**DOI:** 10.3389/fendo.2023.1253903

**Published:** 2023-09-14

**Authors:** Jung-Hyun Park, Moo-Seok Park, Hyung-Jun Kim, Heajung Lee, Jin-Woo Kim, Tae-Jin Song

**Affiliations:** ^1^Department of Oral and Maxillofacial Surgery, Mokdong Hospital, College of Medicine, Ewha Womans University, Seoul, Republic of Korea; ^2^Department of Neurology, Seoul Hospital, Ewha Womans University College of Medicine, Seoul, Republic of Korea

**Keywords:** periodontitis, oral hygiene, tooth brushing, osteoporotic fracture, epidemiology

## Abstract

**Background:**

The aim of this study was to examine the longitudinal association between oral health parameters and osteoporotic fracture.

**Methods:**

The study included participants who received oral health screening by dentists from the National Health Screening cohort database of Korea between 2003 and 2006. The primary outcome was osteoporotic fracture occurrence, which was defined using specific international classification of diseases-10 codes; vertebral fracture (S22.0, S22.1, S32.0, S32.7, T08, M48.4, M48.5, and M49.5), hip fracture (S72.0 and S72.1), distal radius fracture (S52.5 and S52.6), and humerus fracture (S42.2 and S42.3). The presence of periodontitis and various oral health examination findings, such as missing teeth, caries, frequency of tooth brushing, and dental scaling, were analyzed using a Cox proportional hazard model to assess their association with osteoporotic fracture occurrence.

**Results:**

The analysis included a total of 194,192 participants, among whom 16,683 (8.59%) developed osteoporotic fracture during a median follow-up of 10.3 years. Poor oral health status, including periodontitis (adjusted hazard ratio [aHR]: 1.09, 95% confidence interval [CI]: 1.01–1.18, p = 0.039), a higher number of missing teeth (≥15; aHR: 1.59, 95% CI: 1.45–1.75, p < 0.001), and dental caries (≥6; aHR: 1.17, 95% CI: 1.02–1.35, p = 0.030), was associated with an increased risk of osteoporotic fracture. On the other hand, better oral hygiene behaviors such as brushing teeth frequently (≥3 times per day; aHR: 0.82, 95% CI: 0.78–0.86, p < 0.001) and having dental scaling within 1 year (aHR: 0.87, 95% CI: 0.84–0.90, p < 0.001) were negatively associated with the occurrence of osteoporotic fracture.

**Conclusion:**

The study found that poor oral health, such as periodontitis, missing teeth, and dental caries, was associated with an increased risk of osteoporotic fracture. Conversely, good oral hygiene behaviors like frequent teeth brushing and dental scaling within 1 year were associated with a reduced risk. Further research is needed to confirm this association.

## Introduction

Oral health issues including periodontitis, dental caries, and tooth loss are common health concerns that affect a large portion of the population (1, 2). Poor oral health conditions not only have negative impacts on oral health but also exhibit a systematic association with or can potentially trigger the onset of a range of diseases in the human body (3, 4). As an instance, periodontitis can cause both local and systemic inflammation, not only in the oral cavity but throughout the human body. Various systemic diseases, including cardiovascular disease, diabetes, neurodegenerative diseases, and certain cancers, have been found to have a higher incidence among individuals with poor oral health, including tooth loss and dental caries (4–9).

Osteoporotic fractures are a common and serious health concern for humans, with incidence rates increasing with age and exponentially rising in accordance with the global demographic shift towards an aging society (10). These fractures can cause disability and have a significant economic burden on society (10, 11). Hip and spinal fractures are particularly severe, and can result in mortality for those affected (12). Risk factors for osteoporotic fracture include premature menopause, low body mass index, smoking, alcohol, physical inactivity, steroid use, and vitamin D insufficiency. However, further research is needed to identify modifiable or preventable risk factors and associations for osteoporotic fractures (13). For example, systemic inflammatory reactions and diseases such as rheumatoid arthritis are closely linked to osteoporotic fractures (14, 15).

Systemic inflammation resulting from impaired oral health status can contribute to the development of diseases in distant organs. Oral health problems like periodontitis, dental caries, and tooth loss are closely linked to systemic inflammation, which may suggest a potential link between oral health and osteoporotic fractures. We hypothesized that a correlation might exist between poor oral health status and an increased risk of osteoporotic fracture, and conversely, better oral hygiene practices could be associated with a lower risk of osteoporotic fracture. Therefore, we conducted a longitudinal study using a nationwide population-based cohort database to explore the association between oral health examination results and the incidence of osteoporotic fractures.

## Methods

### Data source

This study utilized the NHIS-HEALS (National Health Insurance Service-National Health Screening) cohort database from Korea, which is a government-administered and supported system. The NHIS is the primary insurance provider in Korea, covering almost 97% of the population, while the remaining 3% are supported by the Medical Aid program administered by the NHIS (16–18). Standardized health screenings are recommended for NHIS subscribers every 1-2 years. Around 510,000 individuals between the ages of 40 and 79, representing approximately 10% of the total population, were included in the NIHS-HEALS cohort as they participated in health screenings (19, 20). Individual health screening data, such as weight, height, blood pressure, and laboratory test results, are included in the NIHS-HEALS cohort database. Additionally, the database contains demographic and socioeconomic information, as well as diagnosis, prescription, and treatment claim data. During the health screening process, lifestyle questionnaires are administered, which inquire about oral hygiene behaviors such as tooth brushing frequency and annual dental visits. The examination by a dentist also includes an evaluation of dental health problems, such as dental caries or the number of missing teeth. The analysis was approved by the Institutional Review Board of Ewha Womans University College of Medicine under the reference number 2020-08-018, and a consent waiver was granted for the study. All methods were carried out in accordance with relevant guidelines and regulations that is Declaration of Helsinki.

### Study population

**Figure 1:**
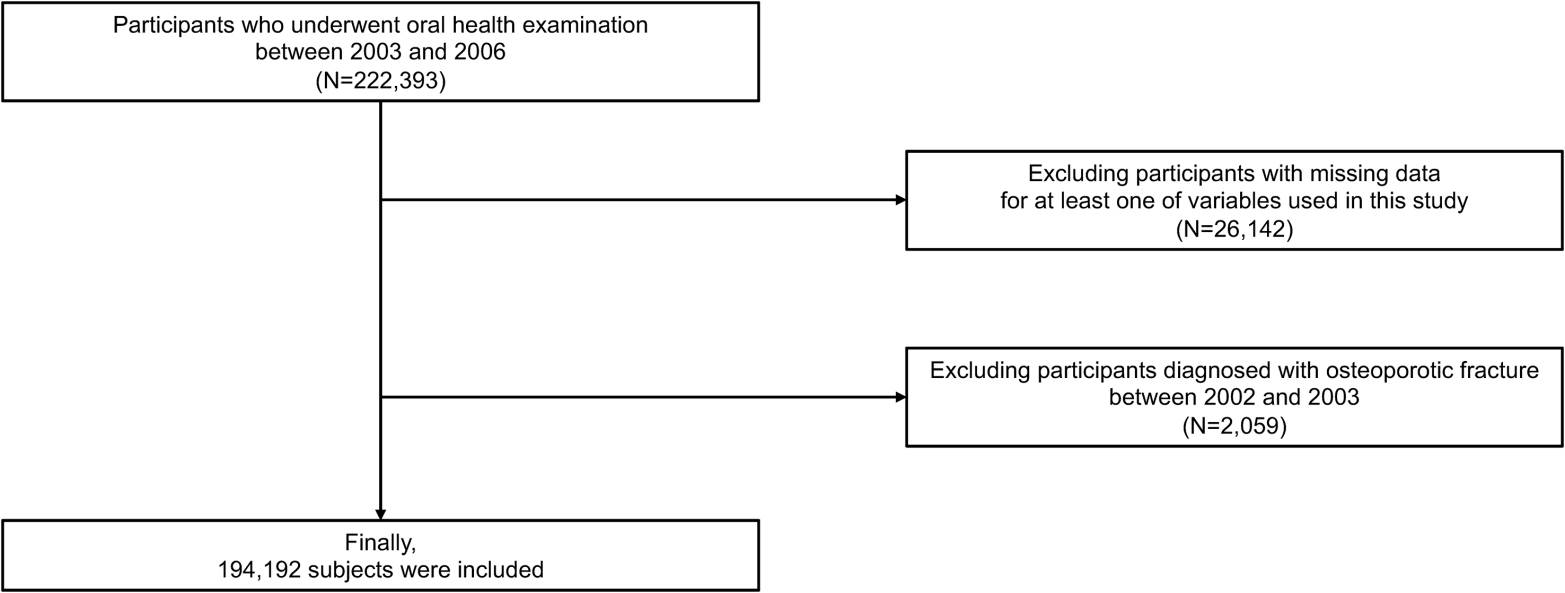
Flow chart of study subjects.

This study utilized the NHIS-HEALS database to identify participants who underwent an oral health examination between 2003 and 2006 (n=222,393). Participants were excluded if data on at least one variable of interest was missing (n=26,142), or if they had a history of osteoporotic fracture from January 2002 until the day before their oral health examination (n=2,059). The final sample included 194,192 participants (**Figure 1**).

### Definition and variables

For this study, the index date was designated as the date of the oral health examination. On this day, baseline characteristics such as age, sex, household income, and body mass index were recorded. Further details about variable definition can be found in the **Supplementary Methods** section.

In order to identify periodontitis in the participants, the period between January 2002 and the index date was evaluated based on the following criteria: 1) two or more claims of ICD-10 codes K052-054 (including acute periodontitis [K052], chronic periodontitis [K053], and periodontitis [K054]) accompanied by at least one claim of treatment codes associated with periodontitis, or 2) identification of a periodontal pocket during oral health examination conducted by a dentist (2, 5). During the oral health examination, a dentist assessed the presence of dental caries and the number of missing teeth. The participants self-reported their oral hygiene behaviors, including the frequency of tooth brushing per day, dental visits within the past year, and dental scaling within the past year. If the oral health examination was conducted more than once, the latest data were used for analysis.

### Study outcomes

The primary outcome of this study was the incidence of osteoporotic fracture. Osteoporotic fracture was defined using ICD-10 codes based on the osteoporotic fracture fact sheet provided by the Korean Society of Bone and Mineral Research. Specifically, it included vertebral fracture (S22.0, S22.1, S32.0, S32.7, T08, M48.4, M48.5, and M49.5), hip fracture (S72.0 and S72.1), distal radius fracture (S52.5 and S52.6), and humerus fracture (S42.2 and S42.3) (12). The follow-up period was from the index date to the occurrence of osteoporotic fracture, death, or December 2015, whichever occurred first, using the NHIS-HEALS database.

### Statistical analysis

To assess the differences in baseline characteristics between the groups, the chi-square test was used for categorical variables and the independent t-test was used for continuous variables. The categorical variables were reported as percentages and numbers, while the continuous variables were reported as mean and standard deviation. To explore the potential link between periodontitis and osteoporotic fracture, we conducted propensity score matching (PSM) with a 1:1 ratio using the greedy nearest-neighbor algorithm to balance the baseline characteristics between the periodontitis and non-periodontitis groups and minimize potential confounding. We used standardized mean differences to assess the appropriateness of PSM, and considered values less than 0.1 to indicate appropriate balance between the two groups. To evaluate the relationship between oral health parameters and the risk of incident osteoporotic fracture, Kaplan-Meier survival curves were utilized. The survival curves were compared using the log-rank test. The incidence rate of osteoporotic fractures was calculated by dividing the number of cases by the total person-years of observation. To examine the potential relationship between oral health parameters with the incidence of osteoporotic fractures, we employed Cox’s proportional hazard regression analysis, and determined hazard ratios (HRs) and their corresponding 95% confidence intervals (CIs). A multivariable regression model was constructed adjusting for age, sex, body mass index, household income, smoking status, alcohol consumption, regular physical activity, and comorbidities such as hypertension, diabetes mellitus, dyslipidemia, atrial fibrillation, and renal disease. We conducted subgroup analyses to investigate the association between the presence of periodontitis and osteoporotic fracture occurrence according to age, sex, and covariates. To assess the robustness of the findings, we conducted sensitivity analyses for each type of osteoporotic fracture (vertebral, hip, distal radius, and humerus fractures). The proportional hazards assumption was checked using Schoenfeld’s residuals, and no violations were detected. Statistical analysis was performed using SAS software (version 9.2, SAS Institute, Cary, NC). A significance level of p < 0.05 was used for all analyses.

## Results

**Table 1** displays the baseline characteristics and comparative analysis of the included participants with respect to the presence of periodontitis. Out of a total of 194,192 participants, 58.9% were male, and the average age was 53.6 ± 8.7 years. Among the participants, 1.1% had more than 15 missing teeth, 1.0% had six or more caries, and 37.1% brushed their teeth more than thrice a day. **Supplementary Table 1** presents the baseline characteristics after performing propensity score matching.

**Table 1:** Baseline characteristics of participants according to periodontitis.

Variable	Total	Periodontitis (-)	Periodontitis (+)	p-value
No. of participants (%)	194192	187748 (96.7)	6444 (3.3)	
Age, years	53.62 ± 8.65	53.57 ± 8.64	55.05 ± 8.87	<.001
Sex				<.001
Male	114275 (58.9)	110025 (58.6)	4250 (66.0)	
Female	79917 (41.2)	77723 (41.4)	2194 (34.0)	
Body mass index (kg/m^2^)	23.94 ± 2.87	23.94 ± 2.87	23.96 ± 2.97	0.642
Household income				<.001
T1, lowest	50489 (26.0)	48635 (25.9)	1854 (28.8)	
T2	66447 (34.2)	63975 (34.1)	2472 (38.4)	
T3, highest	77256 (39.8)	75138 (40.0)	2118 (32.9)	
Smoking status				<.001
Never	132224 (68.1)	128313 (68.3)	3911 (60.7)	
Former	19220 (9.9)	18466 (9.8)	754 (11.7)	
Current	42748 (22.0)	40969 (21.8)	1779 (27.6)	
Alcohol consumption (days/week)				<.001
None	139155 (71.7)	134831 (71.8)	4324 (67.1)	
1–4	48224 (24.8)	46453 (24.7)	1771 (27.5)	
≥5	6813 (3.5)	6464 (3.4)	349 (5.4)	
Regular physical activity (days/week)				<.001
None	94921 (48.9)	91647 (48.8)	3274 (50.8)	
1–4	79900 (41.1)	77428 (41.2)	2472 (38.4)	
≥5	19371 (10.0)	18673 (10.0)	698 (10.8)	
Comorbidities				
Hypertension	54097 (27.9)	52044 (27.7)	2053 (31.9)	<.001
Diabetes mellitus	23649 (12.2)	22661 (12.1)	988 (15.3)	<.001
Dyslipidemia	35126 (18.1)	33969 (18.1)	1157 (18.0)	0.777
Atrial fibrillation	611 (0.3)	591 (0.3)	20 (0.3)	0.950
Renal disease	1841 (1.0)	1775 (1.0)	66 (1.0)	0.521
Oral healtd status				
Number of missing teetd				<.001
0	146389 (75.4)	142460 (75.9)	3929 (61.0)	
1–7	42862 (22.1)	40653 (21.7)	2209 (34.3)	
8–14	2887 (1.5)	2657 (1.4)	230 (3.6)	
≥15	2054 (1.1)	1978 (1.1)	76 (1.2)	
Number of dental caries				<.001
0	158710 (81.7)	153980 (82.0)	4730 (73.4)	
1–5	33609 (17.3)	32050 (17.1)	1559 (24.2)	
≥6	1873 (1.0)	1718 (0.9)	155 (2.4)	
Oral hygiene behaviors				
Frequency of tootd brushing (times/day)				<.001
0–1	28454 (14.7)	27291 (14.5)	1163 (18.1)	
2	93642 (48.2)	90352 (48.1)	3290 (51.1)	
≥3	72096 (37.1)	70105 (37.3)	1991 (30.9)	
Dental visit for any reason				0.684
No	109239 (56.3)	105630 (56.3)	3609 (56.0)	
Yes	84953 (43.8)	82118 (43.7)	2835 (44.0)	
Dental scaling				<.001
No	145614 (75.0)	140534 (74.9)	5080 (78.8)	
Yes	48578 (25.0)	47214 (25.2)	1364 (21.2)	

T, Tertile.

P-value by the Chi-square test for categorical variables and independent t-test for continuous variables. Data are expressed as the mean ± SD, or n (%).

**Figure 2:**
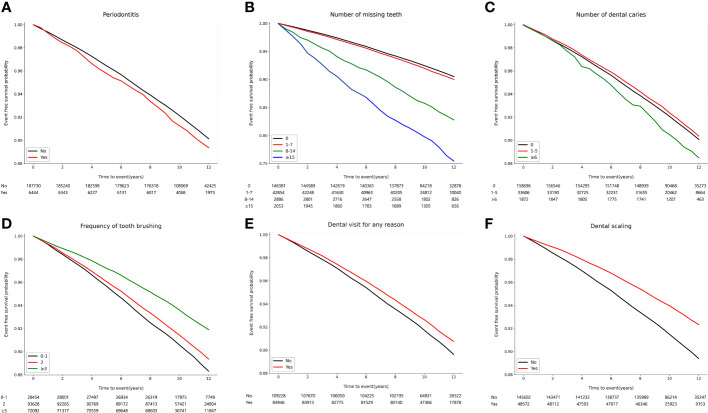
Kaplan–Meier survival curves for incident osteoporotic fracture according to oral health status and oral hygiene behaviors. **(A)** Periodontitis (p = 0.019). **(B)** Number of missing teeth (p < 0.001). **(C)** Number of dental caries (p = 0.008). **(D)** Frequency of tooth brushing (times/per day) (p < 0.001). **(E)** Dental visit for any reason within the past year (p < 0.001). **(F)** Dental scaling within the past year (p < 0.001).

Over a median follow-up of 10.3 years (interquartile range, 9.5-11.6 years), 16,683 (8.6%) participants developed osteoporotic fractures. Among these, there were 7,827 (4.0%) vertebral fractures, 1,866 (1.0%) hip fractures, 7,563 (3.9%) distal radius fractures, and 1,069 (0.6%) humerus fractures. The association of oral health parameters with osteoporotic fracture risk was evaluated using Kaplan-Meier survival curves, as shown in **Figure 2**. The incidence of osteoporotic fracture was significantly higher among participants with periodontitis (p = 0.019), increased number of missing teeth (p < 0.001), and dental caries (p = 0.021). a lower incidence of osteoporotic fracture was observed in individuals with better oral hygiene behaviors, such as brushing teeth more frequently, having visited a dental clinic within the past year, and having received dental scaling within the past year (p < 0.001; **Supplementary Table 2**).

In the multivariable analysis, a positive correlation was found between the presence of periodontitis and the occurrence of osteoporotic fracture (adjusted HR: 1.09, 95% CI: 1.01–1.18, p = 0.039) (**Table 2**). Furthermore, after PSM, the association between periodontitis and the occurrence of osteoporotic fractures remained significant (adjusted HR: 1.04, 95% CI: 1.01–1.06, p = 0.017). In our analysis, we found that an increased number of missing teeth was linked to a higher risk of osteoporotic fractures. The adjusted hazard ratios (with reference to individuals with no missing teeth) were 1.32 (95% CI: 1.20-1.45, p < 0.001) for those with 8-14 missing teeth and 1.59 (95% CI: 1.45-1.75, p < 0.001) for those with more than 15 missing teeth. The occurrence of osteoporotic fracture was found to be positively associated with an increased number of dental caries. Participants with more than six dental caries had an adjusted HR of 1.17 (95% CI: 1.02–1.35, p = 0.030) compared to those without dental caries. Additionally, there was an inverse correlation between the frequency of tooth brushing and the occurrence of osteoporotic fracture. Participants who brushed their teeth twice a day (adjusted HR: 0.90, 95% CI: 0.86–0.94, p < 0.001) and more than thrice a day (adjusted HR: 0.82, 95% CI: 0.78–0.86, p < 0.001) had a reduced risk for developing osteoporotic fractures compared to those who brushed less than once a day. Additionally, participants who had undergone dental scaling within the past year had a statistically significant decrease in the risk of osteoporotic fracture (adjusted HR: 0.87, 95% CI: 0.84–0.90, p < 0.001). Subgroup analysis demonstrated that the association between the presence of periodontitis and the occurrence of osteoporotic fracture remained consistent across all covariates (**Supplementary Table 3**).

**Table 2:** Risk for the occurrence of osteoporotic fracture according to oral health status and oral hygiene behaviors.

	N of participants	N of events	Event rate (%) (95% CI)	Person-years	Incidence rate (per 1000 person-years)	Adjusted HR* (95% CI)	p-value
Oral health status							
Periodontitis							
No	187748	16063	8.56 (8.42, 8.69)	1923793	8.35	1 (reference)	
Yes	6444	620	9.62 (8.86, 10.38)	67099	9.24	**1.09** **(1.01, 1.18)**	**0.039**
Number of missing teeth							
0	146389	12015	8.21 (8.06, 8.35)	1501633	8.00	1 (reference)	
1-7	42862	3748	8.74 (8.46, 9.02)	439785	8.52	1.01 (0.97, 1.05)	0.571
8-14	2887	454	15.73 (14.28, 17.17)	29250	15.52	**1.32** **(1.20, 1.45)**	**<.001**
≥15	2054	466	22.69 (20.63, 24.75)	20224	23.04	**1.59** **(1.45, 1.75)**	**<.001**
Number of dental caries							
0	158710	13662	8.61 (8.46, 8.75)	1622961	8.42	1 (reference)	
1-5	33609	2827	8.41 (8.10, 8.72)	348522	8.11	0.99 (0.95, 1.03)	0.463
≥6	1873	194	10.36 (8.90, 11.82)	19409	10.00	**1.17** **(1.02, 1.35)**	**0.030**
Oral hygiene behaviors							
Frequency of tooth brushing (times/day)							
0-1	28454	2962	10.41 (10.03, 10.78)	294105	10.07	1 (reference)	
2	93642	8804	9.40 (9.21, 9.60)	966415	9.11	**0.90** **(0.86, 0.94)**	**<.001**
≥3	72096	4917	6.82 (6.63, 7.01)	730372	6.73	**0.82** **(0.78, 0.86)**	**<.001**
Dental visit for any reason							
No	109239	9890	9.05 (8.88, 9.23)	1123052	8.81	1 (reference)	
Yes	84953	6793	8.00 (7.81, 8.19)	867841	7.83	**0.97** **(0.94, 1.00)**	**0.043**
Dental scaling							
No	145614	13520	9.28 (9.13, 9.44)	1495153	9.04	1 (reference)	
Yes	48578	3163	6.51 (6.28, 6.74)	495740	6.38	**0.87** **(0.84, 0.90)**	**<.001**

*Adjusted for age, sex, body mass index, household income, smoking status, alcohol consumption, regular physical activity, hypertension, diabetes mellitus, dyslipidemia, atrial fibrillation, and renal disease.

HR, hazard ratio; CI, confidence interval.

Bold indicates statistically significant differences via Cox’s proportional hazard regression analysis.

In the sensitivity analysis, we consistently observed a correlation between oral health status and oral hygiene behaviors (excluding dental caries for vertebral fracture and the presence of periodontitis for hip fracture) and the occurrence of vertebral and hip fractures, similar to that of osteoporotic fracture (**Supplementary Tables 4, 5**). However, only frequent tooth brushing (more than thrice per day) was found to be associated with a reduced risk of distal radius fracture (**Supplementary Table 6**). In addition, we observed that an increased number of tooth loss was related to an increased risk of humerus fracture, while frequent tooth brushing was related to a decreased risk of humerus fracture (**Supplementary Table 7**).

## Discussion

Our study’s main results showed that unfavorable oral health conditions, including periodontitis and a higher number of missing teeth, were linked with an elevated risk of osteoporotic fractures. In contrast, improved oral hygiene practices such as more frequent tooth brushing and previous dental scaling were associated with a decreased risk of osteoporotic fractures.

The systemic consequences of periodontitis and poor oral health are well documented, and various systemic diseases have been associated with them. For example, studies have shown that periodontitis is linked to an increased risk of cardiovascular disease and diabetes (2, 21, 22). Similarly, tooth loss, which is indicative of poor oral health, has been associated with a higher risk of cardiovascular diseases and hypertension (23, 24). Periodontitis or poor oral health status is also likely related to osteoporosis/osteoporotic fracture. Periodontitis is positively associated with osteoporosis, and the level of osteoporosis increases as the severity of periodontitis increases (25–27). Moedano et al. (28) assessed the association of osteoporosis, vertebral fracture risk, and periodontitis and found that periodontitis is positively associated with the severity of osteoporosis; furthermore, patients at a high risk of vertebral fractures have more missing teeth than those in the low-fracture risk group.

On the other hand, behaviors that reduce oral inflammation, such as regular tooth brushing and professional dental care, have been shown to decrease the risk of some systemic diseases. For example, increased frequency of tooth brushing has been associated with a lower risk of strok (7), atrial fibrillation, and heart failure (1). Regular dental check-ups and professional cleaning have also been found to attenuate the risk of cardiovascular events (24). In addition, oral hygiene care is potentially related to a lower risk of osteoporotic fracture. Huang et al. (29) demonstrated a 1.29-fold higher risk of osteoporosis in patients who have periodontitis and receive regular dental treatment compared with those who have no periodontitis. However, the risk for patients who have periodontitis and do not receive dental treatment is 6.02-fold higher than those who have no periodontitis. The study recommended that good oral hygiene should be maintained to prevent the deterioration of osteoporosis.

Most cross-sectional studies have been performed to demonstrate the association between osteoporosis and periodontitis. However, further information has yet to be obtained to describe whether periodontitis increases the risk of future osteoporotic fractures. Therefore, our study aimed to investigate the effect of oral health status by longitudinally tracking the occurrence of osteoporotic fractures. Our findings are significant since they indicate a clear association between oral health and the future risk of osteoporotic fractures. Additionally, our results suggest that preventing periodontitis and chronic oral inflammation through regular dental scaling and maintaining good oral hygiene practices could potentially lower the risk of future osteoporotic fractures.

Despite being unable to establish a direct causal relationship between poor oral health and the occurrence of osteoporotic fractures in our study, we propose a hypothesis to explain the correlation. Periodontitis is a chronic inflammatory disease caused by the accumulation of bacterial biofilm and immune responses. Other chronic inflammatory disorders, including inflammatory bowel disease and rheumatoid arthritis, have been linked to osteoporosis and an elevated risk of fractures (30). The mechanisms underlying the association between chronic inflammatory diseases and osteoporosis may also apply to the relationship between oral inflammation and osteoporosis/osteoporotic fracture.

The pathway of RANK (receptor activator of nuclear factor kappa B) – RANKL (RANK ligand) – OPG (osteoprotegerin) is recognized for its function in bone remodeling, where RANKL/RANK signaling controls the formation, activation, and survival of osteoclasts during regular bone modeling and remodeling. OPG inhibits osteoclast activation through the RANK receptor by binding to RANKL and increases bone volume and density (31, 32). Oral inflammation produces pro-inflammatory cytokines, which are osteoclastogenic bone resorption-inducing cytokines. These inflammatory cytokines upregulate RANKL to activate bone resorption and likely contribute to osteoclast generation from osteoclast progenitors (33, 34). Previous research has shown that patients with periodontitis have lower plasma OPG levels and an increased RANKL/OPG ratio (35, 36), which may contribute to increased systemic bone resorption. T cells triggered by periodontitis could also be implicated in systemic bone resorption. These cells act as regulators of bone turnover in various diseases, including periodontal disease (37), activating macrophages, indirectly activating osteoclasts and their precursors, and directly expressing RANKL (38). Brunetti et al. (37) demonstrated that RANKL is overexpressed in T lymphocyte samples from patients affected by periodontitis compared with that from healthy controls.

In our sensitivity analysis, we found that the occurrence of vertebral and hip fractures showed consistent results with the overall osteoporotic fractures according to oral health indicators. However, the risk of distal radius and humerus fractures based on oral health indicators did not yield consistent results. This study could not explain why the influence of oral health indicators was different in each fracture site. Future studies should aim to clarify the biological mechanisms that account for these regional differences. However, our results may suggest that periodontitis and oral health indicators may be related to each fracture sites through different mechanisms.

In the present study, potential confounders were included as covariates in statistical models to adjust for their influence on the main association. Body mass index was included due to its established correlation with fracture risk, particularly in cases of low body mass index (39). Lifestyle factors influencing bone health, such as alcohol consumption, smoking, and physical activity, were also included as risk factors associated with osteoporotic fractures (40). Additionally, we considered comorbidities that could affect the relationship between the oral health and osteoporotic fracture, including hypertension, diabetes, dyslipidemia, atrial fibrillation, and renal disease. Hypertension is associated with compromised calcium metabolism, which may lead to reduced bone density in the elderly due to increased urinary calcium excretion, thereby increasing fracture susceptibility (41). Additionally, both type 1 and type 2 diabetes have been linked to an elevated risk of osteoporotic fractures, attributed to the absence of bone anabolic effects of insulin or potential impairment of bone quality due to antidiabetic drugs (42). Elevated cholesterol levels have been reported to impact cellular function within bone tissue, recent findings suggesting elevated osteoclast activity alongside diminished osteoblast function, which could potentially increase fracture susceptibility (43). The risk of fracture may be elevated due to a stroke caused by atrial fibrillation, and certain medications used in atrial fibrillation management, such as warfarin, have detrimental effects on bone quality and can potentially be associated with fracture risk (44). Disturbances in mineral regulation in renal disease have been suggested to increase fracture susceptibility (45). Nevertheless, even after considering these confounding factors, our main findings showed a significant association between oral health and future osteoporotic fracture.

This study had several limitations. First, there is a possibility of residual confounding factors affecting the development of osteoporotic fractures. Factors such as education level, nutrition, lifestyle, hormone level, blood/urine calcium, menarche, and menopause are important confounding variables that should be taken into consideration in the analysis. Unfortunately, the NHIS-HEALS database did not provide validated information on these variables, some of which may have been considered personally identifiable or sensitive information, so we were unable to include them in our study. Second, although our study aimed to establish an association between osteoporotic fracture and oral health status assessed at a fixed point, but an individual’s oral health status can be influenced by their personal management and dental care. Therefore, future research is warranted to investigate the impact of changing oral health conditions over time on the incidence of osteoporotic fractures. Third, the occurrence of bone fractures shows significant differences by geographical region, the differences are also large within Europe (downward trend between north and south), and accordingly the expected fracture risk differs from region to region and country to country (46, 47). The results of our study may not be generalizable to other races/ethnicities as we only included Korean participants. Fourth, detailed information on attachment loss was not available in the NIHS-HEALS cohort database, limiting our ability to investigate the severity of periodontitis. Fifth, only 3.3% of participants were diagnosed with periodontitis. This low proportion could be due to the strict definition of periodontitis, which required both diagnostic codes and treatment codes to be present, as well as the age range of participants (≥20 years) (48–50). Sixth, The self-reported nature of oral health behaviors in our study questionnaire could have resulted in response bias. Seventh, as our study was retrospective and observational in design, we cannot infer a causal relationship between oral health status and osteoporotic fractures. Nonetheless, our study also had some notable strengths. We utilized extensive long-term nationally representative data to investigate the association between oral health parameters and osteoporotic fractures. Our findings highlight the crucial role of maintaining good oral health in preventing osteoporotic fractures.

## Conclusion

The risk of osteoporotic fracture may be higher in individuals with periodontitis or a higher number of missing teeth, while good oral hygiene practices like tooth brushing and dental scaling may lower the risk of future fractures. Although this study suggests a possible association between oral health and osteoporotic fracture, further research is needed to confirm this relationship.

## Data availability statement

The datasets analyzed during the current study are available in the [National Health Insurance Service-National Health Screening Cohort (NHIS-HEALS) database] repository, [http://nhiss.nhis.or.kr/bd/ab/bdaba021eng.do] (dataset number: NHIS-2021-01-715). While this data is publicly available, access to the data requires approval. Requests for access to NHIS data must be submitted through the National Health Insurance Sharing Service homepage. Access to the database requires submission of a completed application form, research proposal, and application for approval from the institutional review board to the inquiry committee of research support in the NHIS for review.

## Ethics statement

The Institutional Review Board of Ewha Womans University College of Medicine (2020–08–018) approved the analysis and provided a consent waiver as the data were anonymized and freely accessible by the NHIS for study purposes. All methods were carried out in accordance with relevant guidelines and regulations that is Declaration of Helsinki.

## Author contributions

J-HP: Conceptualization, Methodology, Visualization, Writing – original draft. M-SP: Formal Analysis, Methodology, Writing – review & editing. H-JK: Formal Analysis, Methodology, Writing – review & editing. HL: Data curation, Formal Analysis, Methodology, Writing – review & editing. J-WK: Formal Analysis, Funding acquisition, Methodology, Supervision, Writing – review & editing. T-JS: Conceptualization, Data curation, Formal Analysis, Funding acquisition, Methodology, Supervision, Writing – review & editing.
