# Supplementary material for: Better oral hygiene is associated with a reduced risk of osteoporotic fracture: a nationwide cohort study
Source: Front Endocrinol (Lausanne). 2023 Sep 14;14:1253903. doi: 10.3389/fendo.2023.1253903 (PMC10539647; doi:10.3389/fendo.2023.1253903)
Supplement: Supplementary file 1 [file DataSheet_1.docx]

**Supplementary Methods**

Definition of variables

Smoking habits (nonsmoker, former smoker, and current smoker), alcohol consumption (frequency per week), and regular physical exercise (frequency per week) were collected through questionnaires. Comorbidities that had occurred between January 2002 and the index date were also identified. The criteria for defining hypertension in this study were as follows: 1) a prescription for an antihypertensive agent with at least one diagnostic code (ICD-10 I10–15), 2) two or more diagnostic code claims (ICD-10 I10–15), 3) systolic/diastolic blood pressure readings of ≥140/90 mmHg, or 4) self-reported hypertension in the questionnaire. To define diabetes mellitus, the following criteria were used: 1) prescription of an antidiabetic agent with at least one diagnostic code claim (ICD-10 E11–14), 2) two or more claims of diagnostic codes (ICD-10 E11–14), 3) fasting serum glucose level of ≥7.0 mmol/L, or 4) self-reported diabetes mellitus in the questionnaire. The criteria used to define dyslipidemia were as follows: 1) at least one claim of diagnostic codes (ICD-10 E78) with the prescription of a dyslipidemia-related agent, 2) two or more claims of diagnostic codes (ICD-10 E78), or 3) a total cholesterol level of ≥240 mg/dL. Atrial fibrillation was defined as two or more claims of the diagnostic code (ICD-10 I48). Renal disease was defined as two or more claims of diagnostic codes (ICD-10 N17-19, I12-13, E082, E102, E112, and E132), or an estimated glomerular filtration rate of less than 60 mL/min/1.73 m^2^.
