# Supplementary material for: Better oral hygiene is associated with a reduced risk of osteoporotic fracture: a nationwide cohort study
Source: Front Endocrinol (Lausanne). 2023 Sep 14;14:1253903. doi: 10.3389/fendo.2023.1253903 (PMC10539647; doi:10.3389/fendo.2023.1253903)
Supplement: Supplementary file 2 [file Table_1.docx]

**Supplementary tables**

Supplementary Table 1. Baseline characteristics of participants according to periodontitis after propensity score matching.

Supplementary Table 2. Risk factors for the occurrence of osteoporotic fracture.

Supplementary Table 3. Subgroup analysis regarding periodontitis and osteoporotic fracture in association with demographics or comorbidities.

Supplementary Table 4. Risk for the occurrence of vertebral fracture according to oral health status and oral hygiene behaviors

Supplementary Table 5. Risk for the occurrence of hip fracture according to oral health status and oral hygiene behaviors

Supplementary Table 6. Risk for the occurrence of distal radius fracture according to oral health status and oral hygiene behaviors

Supplementary Table 7. Risk for the occurrence of humerus fracture according to oral health status and oral hygiene behaviors

# **Supplementary Table 1. Baseline characteristics of participants according to periodontitis after propensity score matching.**

| Variable | Periodontitis (-) | Periodontitis (+) | standardized mean difference |
| --- | --- | --- | --- |
| No. of participants (%) | 6444 | 6444 |  |
| Age, years | 54.97±8.64 | 55.05±8.87 | 0.014 |
| Sex |  |  | 0.019 |
| Male | 4236 (65.7) | 4250 (66.0) |  |
| Female | 2208 (34.3) | 2194 (34.0) |  |
| Body mass index (kg/m^2^) | 23.87±2.92 | 23.96±2.97 | 0.038 |
| Household income |  |  | 0.025 |
| T1, lowest | 1839 (28.5) | 1854 (28.8) |  |
| T2 | 2452 (38.1) | 2472 (38.4) |  |
| T3, highest | 2153 (33.4) | 2118 (32.9) |  |
| Smoking status |  |  | 0.081 |
| Never | 4021 (62.4) | 3911 (60.7) |  |
| Former | 734 (11.4) | 754 (11.7) |  |
| Current | 1689 (26.2) | 1779 (27.6) |  |
| Alcohol consumption (days/week) |  |  | 0.092 |
| None | 4434 (68.8) | 4324 (67.1) |  |
| 1–4 | 1689 (26.2) | 1771 (27.5) |  |
| ≥5 | 321 (5.0) | 349 (5.4) |  |
| Regular physical activity (days/week) |  |  | 0.074 |
| None | 3231 (50.1) | 3274 (50.8) |  |
| 1–4 | 2501 (38.8) | 2472 (38.4) |  |
| ≥5 | 712 (11.1) | 698 (10.8) |  |
| Comorbidities |  |  |  |
| Hypertension | 2039 (31.6) | 2053 (31.9) | 0.002 |
| Diabetes mellitus | 979 (15.2) | 988 (15.3) | 0.001 |
| Dyslipidemia | 1139 (17.7) | 1157 (18.0) | 0.011 |
| Atrial fibrillation | 18 (0.3) | 20 (0.3) | 0.028 |
| Renal disease | 62 (1.0) | 66 (1.0) | 0.062 |
| Oral health status |  |  |  |
| Number of missing teeth |  |  | 0.093 |
| 0 | 3989 (61.9) | 3929 (61.0) |  |
| 1–7 | 2173 (33.7) | 2209 (34.3) |  |
| 8–14 | 217 (3.4) | 230 (3.6) |  |
| ≥15 | 65 (1.0) | 76 (1.2) |  |
| Number of dental caries |  |  | 0.089 |
| 0 | 4786 (74.2) | 4730 (73.4) |  |
| 1–5 | 1568 (24.3) | 1559 (24.2) |  |
| ≥6 | 90 (1.4) | 155 (2.4) |  |
| Oral hygiene behaviors |  |  |  |
| Frequency of tooth brushing (times/day) |  |  | 0.095 |
| 0–1 | 1042 (16.2) | 1163 (18.1) |  |
| 2 | 3328 (51.7) | 3290 (51.1) |  |
| ≥3 | 2074 (32.2) | 1991 (30.9) |  |
| Dental visit for any reason |  |  | 0.027 |
| No | 3575 (55.5) | 3609 (56.0) |  |
| Yes | 2869 (44.5) | 2835 (44.0) |  |
| Dental scaling |  |  | 0.086 |
| No | 5045 (78.3) | 5080 (78.8) |  |
| Yes | 1399 (21.7) | 1364 (21.2) |  |

# **Supplementary Table 2. Risk factors for the occurrence of osteoporotic fracture.**

| Variable | Crude HR (95% CI) | p-value | Adjusted HR* (95% CI) | p-value |
| --- | --- | --- | --- | --- |
| Age, years |  |  |  |  |
| <60 | 1 (reference) |  | 1 (reference) |  |
| ≥60 | **3.25 (3.16, 3.35)** | **<.001** | **2.89 (2.79, 2.98)** | **<.001** |
| Sex |  |  |  |  |
| Male | 1 (reference) |  | 1 (reference) |  |
| Female | **3.06 (2.97, 3.16)** | **<.001** | **2.94 (2.84, 3.04)** | **<.001** |
| Body mass index (kg/m^2^) | **0.98 (0.98, 0.99)** | **<.001** | **0.97 (0.96, 0.98)** | **<.001** |
| Household income |  |  |  |  |
| T1, lowest | 1 (reference) |  | 1 (reference) |  |
| T2 | **0.79 (0.76, 0.82)** | **<.001** | **0.96 (0.93, 0.99)** | **0.039** |
| T3, highest | **0.61 (0.59, 0.63)** | **<.001** | **0.90 (0.86, 0.94)** | **<.001** |
| Smoking |  |  |  |  |
| Never | 1 (reference) |  | 1 (reference) |  |
| Former | **0.42 (0.40, 0.45)** | **<.001** | 0.95 (0.87, 1.03) | 0.194 |
| Current | **0.49 (0.47, 0.52)** | **<.001** | **1.08 (1.04, 1.12)** | **0.003** |
| Alcohol consumption (days/week) |  |  |  |  |
| <1 | 1 (reference) |  | 1 (reference) |  |
| 1–4 | **0.50 (0.48, 0.52)** | **<.001** | 0.99 (0.94, 1.03) | 0.671 |
| ≥5 | **0.86 (0.79, 0.93)** | **<.001** | **1.28 (1.18, 1.38)** | **<.001** |
| Regular physical activity (days/week) |  |  |  |  |
| <1 | 1 (reference) |  | 1 (reference) |  |
| 1–4 | **0.60 (0.58, 0.62)** | **<.001** | **0.83 (0.79, 0.87)** | **<.001** |
| ≥5 | 0.95 (0.91, 1.00) | 0.045 | 0.98 (0.93, 1.03) | 0.273 |
| Comorbidities |  |  |  |  |
| Hypertension | **1.44 (1.39, 1.48)** | **<.001** | **1.16 (1.12, 1.20)** | **<.001** |
| Diabetes mellitus | **1.32 (1.27, 1.38)** | **<.001** | **1.16 (1.11, 1.21)** | **<.001** |
| Dyslipidemia | **1.25 (1.21, 1.30)** | **<.001** | 0.99 (0.95, 1.03) | 0.544 |
| Atrial fibrillation | 1.21 (0.95, 1.55) | 0.123 | 0.95 (0.74, 1.22) | 0.686 |
| Renal disease | **1.40 (1.23, 1.60)** | **<.001** | 0.99 (0.86, 1.13) | 0.854 |
| Oral health status |  |  |  |  |
| Periodontitis |  |  |  |  |
| No | 1 (reference) |  | 1 (reference) |  |
| Yes | **1.10 (1.02, 1.19)** | **0.019** | **1.09 (1.01, 1.18)** | **0.037** |
| Number of missing teeth |  |  |  |  |
| 0 | 1 (reference) |  | 1 (reference) |  |
| 1–7 | **1.06 (1.03, 1.10)** | **<.001** | 1.01 (0.97, 1.05) | 0.573 |
| 8–14 | **1.93 (1.76, 2.12)** | **<.001** | **1.32 (1.20, 1.44)** | **<.001** |
| ≥15 | **2.86 (2.61, 3.14)** | **<.001** | **1.58 (1.44, 1.73)** | **<.001** |
| Number of dental caries |  |  |  |  |
| 0 | 1 (reference) |  | 1 (reference) |  |
| 1–5 | 0.96 (0.92, 1.00) | 0.050 | 0.99 (0.95, 1.03) | 0.465 |
| ≥6 | **1.18 (1.03, 1.36)** | **0.021** | **1.16 (1.02, 1.31)** | **0.032** |
| Oral hygiene behaviors |  |  |  |  |
| Frequency of tooth brushing (times/day) |  |  |  |  |
| 0–1 | 1 (reference) |  | 1 (reference) |  |
| 2 | **0.91 (0.87, 0.94)** | **<.001** | **0.90 (0.86, 0.94)** | **<.001** |
| ≥3 | **0.67 (0.64, 0.71)** | **<.001** | **0.83 (0.78, 0.87)** | **<.001** |
| Dental visit for any reason |  |  |  |  |
| No | 1 (reference) |  | 1 (reference) |  |
| Yes | **0.89 (0.86, 0.92)** | **<.001** | **0.97 (0.94, 0.99)** | **0.044** |
| Dental scaling |  |  |  |  |
| No | 1 (reference) |  | 1 (reference) |  |
| Yes | **0.71 (0.68, 0.74)** | **<.001** | **0.86 (0.82, 0.90)** | **<.001** |

*Adjusted for age, sex, body mass index, household income, smoking status, alcohol consumption, regular physical activity, hypertension, diabetes mellitus, dyslipidemia, atrial fibrillation, and renal disease.

HR = hazard ratio; CI = confidence interval.

Bold indicates statistically significant differences via Cox’s proportional hazard regression analysis.

# **Supplementary Table 3. Subgroup analysis regarding periodontitis and osteoporotic fracture associated with demographics or comorbidities.**

| Variable | Adjusted HR* (95% CI) | p-value for interaction effect |
| --- | --- | --- |
| Age, years |  | 0.258 |
| <60 | 0.93 (0.77, 1.15) |  |
| ≥60 | 1.13 (1.03, 1.23) |  |
| Sex |  | 0.376 |
| Male | 1.04 (0.92, 1.18) |  |
| Female | 1.11 (1.01, 1.21) |  |
| Body mass index (kg/m^2^) |  | 0.752 |
| <25 | 1.09 (0.99, 1.20) |  |
| ≥25 | 1.09 (0.94, 1.24) |  |
| Household income |  | 0.732 |
| T1, lowest | 1.07 (0.93, 1.21) |  |
| T2 | 1.13 (1.00, 1.27) |  |
| T3, highest | 1.05 (0.89, 1.23) |  |
| Smoking |  | 0.942 |
| Never | 1.09 (0.99, 1.20) |  |
| Former | 1.09 (0.80, 1.45) |  |
| Current | 1.07 (0.88, 1.28) |  |
| Alcohol consumption (days/week) |  | 0.345 |
| <1 | 1.10 (1.01, 1.19) |  |
| 1–4 | 0.97 (0.79, 1.20) |  |
| ≥5 | 1.26 (0.91, 1.72) |  |
| Regular physical activity (days/week) |  | 0.443 |
| <1 | 1.04 (0.94, 1.14) |  |
| 1–4 | 1.15 (0.99, 1.32) |  |
| ≥5 | 1.21 (0.97, 1.44) |  |
| Comorbidities |  |  |
| Hypertension |  | 0.109 |
| No | 1.18 (1.07, 1.29) |  |
| Yes | 0.94 (0.81, 1.07) |  |
| Diabetes mellitus |  | 0.323 |
| No | 1.07 (0.97, 1.17) |  |
| Yes | 1.21 (1.00, 1.43) |  |
| Dyslipidemia |  | 0.219 |
| No | 1.11 (1.02, 1.20) |  |
| Yes | 0.99 (0.82, 1.16) |  |
| Atrial fibrillation |  | 0.993 |
| No | 1.09 (1.00, 1.18) |  |
| Yes | 1.00 (0.24, 4.14) |  |
| Renal disease |  | 0.764 |
| No | 1.09 (1.00, 1.18) |  |
| Yes | 1.17 (0.60, 2.28) |  |

*Adjusted for age, sex, body mass index, household income, smoking status, alcohol consumption, regular physical activity, hypertension, diabetes mellitus, dyslipidemia, atrial fibrillation, and renal disease.

HR = hazard ratio; CI = confidence interval.

# **Supplementary Table 4. Risk for the occurrence of vertebral fracture according to oral health status and oral hygiene behaviors.**

|  | N of participants | N of events | Event rate (%) (95% CI) | Person-years | Incidence rate (per 1000 person-years) | Adjusted HR* (95% CI) | p-value |
| --- | --- | --- | --- | --- | --- | --- | --- |
| Oral health status |  |  |  |  |  |  |  |
| Periodontitis |  |  |  |  |  |  |  |
| No | 187748 | 7476 | 3.98 (3.89, 4.07) | 1911194 | 3.91 | 1 (reference) |  |
| Yes | 6444 | 351 | 5.45 (4.88, 6.02) | 65617 | 5.35 | **1.29 (1.15, 1.43)** | **<.001** |
| Number of missing teeth |  |  |  |  |  |  |  |
| 0 | 146389 | 5446 | 3.72 (3.62, 3.82) | 1496117 | 3.64 | 1 (reference) |  |
| 1–7 | 42862 | 1840 | 4.29 (4.10, 4.49) | 434329 | 4.24 | 1.04 (0.98, 1.10) | 0.168 |
| 8–14 | 2887 | 256 | 8.87 (7.78, 9.95) | 27857 | 9.19 | **1.46 (1.29, 1.66)** | **<.001** |
| ≥15 | 2054 | 285 | 13.88 (12.26, 15.49) | 18508 | 15.40 | **1.93 (1.71, 2.18)** | **<.001** |
| Number of dental caries |  |  |  |  |  |  |  |
| 0 | 158710 | 6435 | 4.05 (3.96, 4.15) | 1613398 | 3.99 | 1 (reference) |  |
| 1–5 | 33609 | 1301 | 3.87 (3.66, 4.08) | 344691 | 3.77 | 0.97 (0.91, 1.03) | 0.309 |
| ≥6 | 1873 | 91 | 4.86 (3.86, 5.86) | 18723 | 4.86 | 1.18 (0.96, 1.45) | 0.126 |
| Oral hygiene behaviors |  |  |  |  |  |  |  |
| Frequency of tooth brushing (times/day) |  |  |  |  |  |  |  |
| 0–1 | 28454 | 1516 | 5.33 (5.06, 5.60) | 286619 | 5.29 | 1 (reference) |  |
| 2 | 93642 | 4229 | 4.52 (4.38, 4.65) | 960884 | 4.40 | **0.88 (0.83, 0.93)** | **<.001** |
| ≥3 | 72096 | 2082 | 2.89 (2.76, 3.01) | 729308 | 2.86 | **0.76 (0.71, 0.81)** | **<.001** |
| Dental visit for any reason |  |  |  |  |  |  |  |
| No | 109239 | 4769 | 4.37 (4.24, 4.49) | 1111634 | 4.29 | 1 (reference) |  |
| Yes | 84953 | 3058 | 3.60 (3.47, 3.73) | 865177 | 3.54 | **0.91 (0.87, 0.96)** | **<.001** |
| Dental scaling |  |  |  |  |  |  |  |
| No | 145614 | 6522 | 4.48 (4.37, 4.59) | 1481316 | 4.40 | 1 (reference) |  |
| Yes | 48578 | 1305 | 2.69 (2.54, 2.83) | 495495 | 2.63 | **0.78 (0.74, 0.83)** | **<.001** |

*Adjusted for age, sex, body mass index, household income, smoking status, alcohol consumption, regular physical activity, hypertension, diabetes mellitus, dyslipidemia, atrial fibrillation, and renal disease.

HR = hazard ratio; CI = confidence interval.

Bold indicates statistically significant differences via Cox’s proportional hazard regression analysis.

# **Supplementary Table 5. Risk for the occurrence of hip fracture according to oral health status and oral hygiene behaviors.**

|  | N of participants | N of events | Event rate (%) (95% CI) | Person-years | Incidence rate (per 1000 person-years) | Adjusted HR* (95% CI) | p-value |
| --- | --- | --- | --- | --- | --- | --- | --- |
| Oral health status |  |  |  |  |  |  |  |
| Periodontitis |  |  |  |  |  |  |  |
| No | 187748 | 1782 | 0.95 (0.91, 0.99) | 1940027 | 0.92 | 1 (reference) |  |
| Yes | 6444 | 84 | 1.30 (1.02, 1.58) | 66994 | 1.25 | 1.18 (0.95, 1.47) | 0.134 |
| Number of missing teeth |  |  |  |  |  |  |  |
| 0 | 146389 | 1211 | 0.83 (0.78, 0.87) | 1517312 | 0.80 | 1 (reference) |  |
| 1–7 | 42862 | 463 | 1.08 (0.98, 1.18) | 441128 | 1.05 | **1.13 (1.02, 1.26)** | **0.024** |
| 8–14 | 2887 | 83 | 2.87 (2.26, 3.49) | 28918 | 2.87 | **1.95 (1.56, 2.43)** | **<.001** |
| ≥15 | 2054 | 109 | 5.31 (4.31, 6.30) | 19663 | 5.54 | **2.83 (2.32, 3.46)** | **<.001** |
| Number of dental caries |  |  |  |  |  |  |  |
| 0 | 158710 | 1520 | 0.96 (0.91, 1.01) | 1638504 | 0.93 | 1 (reference) |  |
| 1–5 | 33609 | 317 | 0.94 (0.84, 1.05) | 349496 | 0.91 | 1.00 (0.89, 1.13) | 0.942 |
| ≥6 | 1873 | 29 | 1.55 (0.98, 2.11) | 19021 | 1.53 | **1.48 (1.02, 2.14)** | **0.037** |
| Oral hygiene behaviors |  |  |  |  |  |  |  |
| Frequency of tooth brushing (times/day) |  |  |  |  |  |  |  |
| 0–1 | 28454 | 409 | 1.44 (1.30, 1.58) | 292472 | 1.40 | 1 (reference) |  |
| 2 | 93642 | 957 | 1.02 (0.96, 1.09) | 977776 | 0.98 | **0.81 (0.73, 0.92)** | **<.001** |
| ≥3 | 72096 | 500 | 0.69 (0.63, 0.75) | 736779 | 0.68 | **0.73 (0.64, 0.84)** | **<.001** |
| Dental visit for any reason |  |  |  |  |  |  |  |
| No | 109239 | 1168 | 1.07 (1.01, 1.13) | 1130311 | 1.03 | 1 (reference) |  |
| Yes | 84953 | 698 | 0.82 (0.76, 0.88) | 876710 | 0.80 | **0.84 (0.76, 0.92)** | **<.001** |
| Dental scaling |  |  |  |  |  |  |  |
| No | 145614 | 1616 | 1.11 (1.06, 1.16) | 1506500 | 1.07 | 1 (reference) |  |
| Yes | 48578 | 250 | 0.51 (0.45, 0.58) | 500521 | 0.50 | **0.60 (0.52, 0.68)** | **<.001** |

*Adjusted for age, sex, body mass index, household income, smoking status, alcohol consumption, regular physical activity, hypertension, diabetes mellitus, dyslipidemia, atrial fibrillation, and renal disease.

HR = hazard ratio; CI = confidence interval.

Bold indicates statistically significant differences via Cox’s proportional hazard regression analysis.

# **Supplementary Table 6. Risk for the occurrence of distal radius fracture according to oral health status and oral hygiene behaviors.**

|  | N of participants | N of events | Event rate (%) (95% CI) | Person-years | Incidence rate (per 1000 person-years) | Adjusted HR* (95% CI) | p-value |
| --- | --- | --- | --- | --- | --- | --- | --- |
| Oral health status |  |  |  |  |  |  |  |
| Periodontitis |  |  |  |  |  |  |  |
| No | 187748 | 7336 | 3.91 (3.82, 4.00) | 1910215 | 3.84 | 1 (reference) |  |
| Yes | 6444 | 227 | 3.52 (3.06, 3.98) | 66152 | 3.43 | 0.93 (0.82, 1.06) | 0.295 |
| Number of missing teeth |  |  |  |  |  |  |  |
| 0 | 146389 | 5700 | 3.89 (3.79, 3.99) | 1493609 | 3.82 | 1 (reference) |  |
| 1–7 | 42862 | 1575 | 3.67 (3.49, 3.86) | 435064 | 3.62 | 0.96 (0.91, 1.01) | 0.134 |
| 8–14 | 2887 | 156 | 5.40 (4.56, 6.25) | 28345 | 5.50 | 1.11 (0.95, 1.31) | 0.192 |
| ≥15 | 2054 | 132 | 6.43 (5.33, 7.52) | 19350 | 6.82 | 1.12 (0.94, 1.34) | 0.192 |
| Number of dental caries |  |  |  |  |  |  |  |
| 0 | 158710 | 6218 | 3.92 (3.82, 4.02) | 1613008 | 3.86 | 1 (reference) |  |
| 1–5 | 33609 | 1267 | 3.77 (3.56, 3.98) | 344600 | 3.68 | 0.98 (0.92, 1.04) | 0.420 |
| ≥6 | 1873 | 78 | 4.16 (3.24, 5.09) | 18760 | 4.16 | 1.09 (0.87, 1.36) | 0.457 |
| Oral hygiene behaviors |  |  |  |  |  |  |  |
| Frequency of tooth brushing (times/day) |  |  |  |  |  |  |  |
| 0–1 | 28454 | 1148 | 4.03 (3.80, 4.27) | 288172 | 3.98 | 1 (reference) |  |
| 2 | 93642 | 4010 | 4.28 (4.15, 4.41) | 961094 | 4.17 | 0.97 (0.91, 1.03) | 0.338 |
| ≥3 | 72096 | 2405 | 3.34 (3.20, 3.47) | 727101 | 3.31 | **0.90 (0.84, 0.97)** | **0.003** |
| Dental visit for any reason |  |  |  |  |  |  |  |
| No | 109239 | 4361 | 3.99 (3.87, 4.11) | 1112680 | 3.92 | 1 (reference) |  |
| Yes | 84953 | 3202 | 3.77 (3.64, 3.90) | 863687 | 3.71 | 1.02 (0.98, 1.07) | 0.348 |
| Dental scaling |  |  |  |  |  |  |  |
| No | 145614 | 5949 | 4.09 (3.98, 4.19) | 1482825 | 4.01 | 1 (reference) |  |
| Yes | 48578 | 1614 | 3.32 (3.16, 3.48) | 493543 | 3.27 | 0.97 (0.92, 1.03) | 0.307 |

*Adjusted for age, sex, body mass index, household income, smoking status, alcohol consumption, regular physical activity, hypertension, diabetes mellitus, dyslipidemia, atrial fibrillation, and renal disease.

HR = hazard ratio; CI = confidence interval.

Bold indicates statistically significant differences via Cox’s proportional hazard regression analysis.

# **Supplementary Table 7. Risk for the occurrence of humerus fracture according to oral health status and oral hygiene behaviors.**

|  | N of participants | N of events | Event rate (%) (95% CI) | Person-years | Incidence rate (per 1000 person-years) | Adjusted HR* (95% CI) | p-value |
| --- | --- | --- | --- | --- | --- | --- | --- |
| Oral health status |  |  |  |  |  |  |  |
| Periodontitis |  |  |  |  |  |  |  |
| No | 187748 | 1023 | 0.54 (0.51, 0.58) | 1942061 | 0.53 | 1 (reference) |  |
| Yes | 6444 | 46 | 0.71 (0.51, 0.92) | 67015 | 0.69 | 1.28 (0.95, 1.73) | 0.100 |
| Number of missing teeth |  |  |  |  |  |  |  |
| 0 | 146389 | 771 | 0.53 (0.49, 0.56) | 1518355 | 0.51 | 1 (reference) |  |
| 1–7 | 42862 | 243 | 0.57 (0.50, 0.64) | 441734 | 0.55 | 1.05 (0.91, 1.22) | 0.493 |
| 8–14 | 2887 | 28 | 0.97 (0.61, 1.33) | 29075 | 0.96 | **1.47 (1.01, 2.15)** | **0.047** |
| ≥15 | 2054 | 27 | 1.31 (0.82, 1.81) | 19912 | 1.36 | **1.81 (1.23, 2.67)** | **0.003** |
| Number of dental caries |  |  |  |  |  |  |  |
| 0 | 158710 | 877 | 0.55 (0.52, 0.59) | 1640050 | 0.54 | 1 (reference) |  |
| 1–5 | 33609 | 179 | 0.53 (0.45, 0.61) | 349929 | 0.51 | 0.99 (0.84, 1.17) | 0.923 |
| ≥6 | 1873 | 13 | 0.69 (0.32, 1.07) | 19097 | 0.68 | 1.31 (0.76, 2.27) | 0.328 |
| Oral hygiene behaviors |  |  |  |  |  |  |  |
| Frequency of tooth brushing (times/day) |  |  |  |  |  |  |  |
| 0–1 | 28454 | 197 | 0.69 (0.60, 0.79) | 293019 | 0.67 | 1 (reference) |  |
| 2 | 93642 | 521 | 0.56 (0.51, 0.60) | 978827 | 0.53 | **0.78 (0.67, 0.93)** | **0.004** |
| ≥3 | 72096 | 351 | 0.49 (0.44, 0.54) | 737229 | 0.48 | **0.81 (0.68, 0.97)** | **0.022** |
| Dental visit for any reason |  |  |  |  |  |  |  |
| No | 109239 | 612 | 0.56 (0.52, 0.60) | 1131753 | 0.54 | 1 (reference) |  |
| Yes | 84953 | 457 | 0.54 (0.49, 0.59) | 877323 | 0.52 | 0.99 (0.88, 1.12) | 0.925 |
| Dental scaling |  |  |  |  |  |  |  |
| No | 145614 | 828 | 0.57 (0.53, 0.61) | 1508755 | 0.55 | 1 (reference) |  |
| Yes | 48578 | 241 | 0.50 (0.43, 0.56) | 500321 | 0.48 | 0.98 (0.85, 1.14) | 0.823 |

*Adjusted for age, sex, body mass index, household income, smoking status, alcohol consumption, regular physical activity, hypertension, diabetes mellitus, dyslipidemia, atrial fibrillation, and renal disease.

HR = hazard ratio; CI = confidence interval.

Bold indicates statistically significant differences via Cox’s proportional hazard regression analysis.
